# rKLO8, a Novel *Leishmania donovani* – Derived Recombinant Immunodominant Protein for Sensitive Detection of Visceral Leishmaniasis in Sudan

**DOI:** 10.1371/journal.pntd.0002322

**Published:** 2013-07-18

**Authors:** Elfadil Abass, Nadine Bollig, Katharina Reinhard, Bärbel Camara, Durria Mansour, Alexander Visekruna, Michael Lohoff, Ulrich Steinhoff

**Affiliations:** 1 Institute for Medical Microbiology and Hygiene, University of Marburg, Marburg, Germany; 2 Biomedical Research Laboratory, Ahfad University for Women, Omdurman, Sudan; Royal Tropical Institute, the Netherlands

## Abstract

**Background:**

For effective control of visceral leishmaniasis (VL) in East Africa, new rapid diagnostic tests are required to replace current tests with low sensitivity. The aim of this study is to improve diagnosis of VL in East Africa by testing a new antigen from an autochthonous *L. donovani* strain in Sudan.

**Methodology and Principle Findings:**

We cloned, expressed and purified a novel recombinant protein antigen of *L. donovani* from Sudan, designated rKLO8, that contains putative conserved domains with significant similarity to the immunodominant kinesin proteins of *Leishmania*. rKLO8 exhibited 93% and 88% amino acid identity with cloned kinesin proteins of *L. infantum* (synonymous *L. chagasi*) (K39) and *L. donovani* (KE16), respectively. We evaluated the diagnostic efficiency of the recombinant protein in ELISA for specific detection of VL patients from Sudan. Data were compared with a rK39 ELISA and two commercial kits, the rK39 strip test and the direct agglutination test (DAT). Of 106 parasitologically confirmed VL sera, 104 (98.1%) were tested positive by rKLO8 as compared to 102 (96.2%) by rK39. Importantly, the patients' sera showed increased reactivity with rKLO8 than rK39. Specificity was 96.1% and 94.8% for rKLO8- and rK39 ELISAs, respectively. DAT showed 100% specificity and 94.3% sensitivity while rK39 strip test performed with 81.1% sensitivity and 98.7% specificity.

**Conclusion:**

The increased reactivity of Sudanese VL sera with the rKLO8 makes this antigen a potential candidate for diagnosis of visceral leishmaniasis in Sudan. However, the suitability at the field level will depend on its performance in a rapid test format.

## Author Summary

Visceral leishmaniasis (VL) is an infectious disease caused by the *Leishmania donovani* complex including *Leishmania donovani* in East Africa and India and by *Leishmania infantum* in the Mediterranean area and Latin America. Clinical diagnosis of VL in East Africa is difficult as maladies with similar symptoms are endemic. For this reason, reliable diagnosis of VL is extremely important. However, tests based on antibody reaction with rK39 are not sensitive in East Africa most likely due to the genetic diversity of different *Leishmania* species. In this study, we cloned and expressed a new antigenic protein (rKLO8) of *L. donovani* strain originating from Sudan. Sequence analysis confirmed that KLO8 differs from other kinesin proteins of *Leishmania*. We thus tested and compared the performance of rKLO8 with rK39 and other commercial tests for VL diagnosis in Sudan. Our data show that sera of VL patients reacted stronger with rKLO8 than rK39, suggesting improved diagnosis of patients with low antibody titres.

## Introduction

Visceral leishmaniasis (VL) is a protozoan parasitic diseases caused by members of the *Leishmania donovani* (*L. d*) complex that includes *L. d. donovani* in East Africa and the Indian subcontinent, *L. d. infantum* in Europe and North Africa and *L. d. chagasi* in Latin America [1], [2]. However, recent molecular and enzymatic studies revealed that *L. chagasi* is synonymous with *L. infantum* [3], [4]. Visceral leishmaniasis is still a major health problem with approximately 0.2–0.4 million new cases annually [5]. The majority of infections (90%) occur in countries like India, Bangladesh, Sudan, South Sudan, Brazil and Ethiopia with East African countries having the second highest disease burden after the Indian continent [5].

Sudan has the highest number of reported cases in East Africa [5] where the disease is endemic since the early 1900s, in particular in eastern and central regions [6]. The diagnosis of VL in Sudan is difficult, as the disease is endemic in rural areas with no or little access to medical facilities. Detection of *Leishmania* amastigotes in tissue aspirates is still used for confirmation of the disease in Sudan although it is invasive and of low sensitivity. Diagnosis is further hindered, as the disease sometimes appears with atypical clinical pictures [7] which need confirmation by laboratory tests. Due to high fatality and toxicity of commonly used drugs [8], [9], diagnostic tests have to be of high accuracy.

Commercially available rapid tests are either based on the rK39 of *L. infantum* (synonym. *L. chagasi*) [10] or rKE16 of *L. donovani* [11]. Field tests based on rK39 are used in several countries with high reliability [12]–[17]. However, the low sensitivity in Sudan limits its use in this region [7], [18], [19], [20]. New rapid tests based on the recombinant protein rKE16 from an Indian strain of *L. donovani* have shown similarly high sensitivity and specificity compared to the classically used rK39-based tests in India [11], [21]. A multiregional study with five different rapid tests based on either rK39 or rKE16 demonstrated equal performance with high sensitivity (92.8–100%) in India [20]. However, sensitivity was significantly lower (36.8–92%) in Brazil and East Africa [20].

The direct agglutination test (DAT), which detects antibodies against whole *L. donovani* promastigotes, has proven to be a useful tool for diagnosis of VL in several countries including Sudan [22]–[27]. The stability of DAT was improved by using freeze-dried and glycerol preserved antigens which does not require storage at 4°C thus making the test suitable for field application [28], [29]. However, the test procedure and the need for overnight incubation give limitations for the field use.

Here, we report identification, expression and testing of a new immunodominant protein (rKLo8) from an autochthonous *L. donovani* strain in Sudan. For detection of VL antibodies in patients and controls from Sudan, an rKLo8 ELISA was developed and compared with the rK39 ELISA and two commercial kits.

## Methods

### Parasite and culture

The *L. donovani* reference strain Lo8 was kindly provided by Prof. Bernhard Fleischer, Bernhard Nocht Institute for Tropical Medicine (BNITM), Hamburg. The strain was originally isolated in Sudan from a confirmed case of visceral leishmaniasis. The parasite was maintained in RPMI-1640 supplemented with L-glutamine, NaHCO_3_ (Sigma-Aldrich) and 10% (v/v) fetal calf serum (Sigma-Aldrich).

### Ethical statement

Sera used in this study were collected in the rural hospital of Doka, Eastern State of Sudan [30], [31]. All patients and controls have given consent for participation in the study. Tests and experiments with patients' sera and *Leishmania* strains were anonymized. The study was approved by the Ethical Review Committee of the Federal Ministry of Health in Sudan and by the Regierungspräsidium Gießen, Germany.

### Human sera

A total number of 183 human serum samples were obtained from the serum bank at the Biomedical Research Laboratory, Ahfad University (Omdurman-Sudan). The majority of samples (106) were from VL patients with confirmed lymph-node aspiration, 30 from healthy individuals resident at Doka village (an endemic area for VL) and 20 from healthy people living in the non-endemic area of Omdurman city, 11 from confirmed malaria cases, 10 from patients with diagnosed pulmonary tuberculosis and 6 from leukaemic patients. Diagnosis of VL was done at the rural hospital in Doka by two expert laboratory technicians and sera were collected only from patients older than 2 years. Sera of patients and diseased controls were collected before administration of treatment. All sera were stored frozen (−20°C) at the Biomedical Research Laboratory. Sera from healthy controls were used to determine the ELISA cut off value. Sera were tested blindly without knowing their clinical status or results of lymph node smears.

### Molecular cloning of the *KLO8* gene

The partial gene fragment encoding the immunodominant repeats of *L. donovani*, designated *KLO8*, were amplified from promastigote genomic DNA using the forward (5′-GAGCTCGCAACCGAGTGGGAGG–3′) and reverse (5′- GCTCCGCAGCGCGCTCC–3′) primers, designed according to the published *L. chagasi* gene for kinesin-related protein (GenBank: L07879.1). PCR reaction was performed using Phusion High-Fidelity DNA Polymerase (FINNZYMES OY, Finland) in a total volume of 50 µl, containing 3% (v/v) DMSO, 10 µl HF buffer, 10 mM dNTPs mix-OLS (OMNI life science) and 100 ng genomic DNA. PCR conditions were as follows: denaturation at 98°C for 30 s followed by 25 cycles of denaturation at 98°C for 10 s, annealing at 65°C for 20 s, and extension at 72°C for 20 s. Amplified products revealed multiple bands with sizes equivalent to 117 bp repeats. The largest amplification product (883 bp) was gel purified, digested with *EcoRV* and cloned (according to the manufacturer's instructions) into the plasmid vector pcDNA3.1(+) (Invitrogen life technologies, USA) generating the non-tagged KLO8 construct, pcDNA/KLO8. The sequence was confirmed by restriction digestion with *BamH*I and *Xba*I (Fermentas GmbH, Germany) and by sequence analysis at Seqlab-Sequence Laboratories, Göttingen GmbH. Each insert was sequenced at least twice.

### Expression and purification of the recombinant protein rKLO8 in *E. coli*

For expression and purification of the recombinant protein, *KLO8* was subcloned into the His-tag vector pQE41 (Qiagen GmbH, Germany). The DNA construct pcDNA/KLO8 was used as template with the forward (5′-GTGGAATTCTGCAGATGGATCCATGGAGCTCGCAACC–3′) and reverse (5′-GCCGCCACTGTGCTGGATGTCGACGCTCC–3′) primers, designed to introduce restriction sites for the enzymes *BamH*I and *Sal*I (underlined). Amplification was performed using Phusion Hot Start II DNA Polymerase (Thermofisher Scientific, USA) as recommended by the manufacturer. Amplified DNA fragments were digested with the same restriction enzymes and cloned in-frame and down stream of 6× His-tag into the corresponding sites of the vector pQE41 to generate the plasmid construct carrying the target gene, named as pQE41/KLO8. The recombinant plasmid was verified by DNA sequencing and restriction analysis and then transformed into competent *M15 E.coli* cells (Qiagen GmbH, Germany). *E. coli* were grown at 37°C in Luria-Bertani (LB) medium containing 100 µg/ml ampicillin (Sigma-Aldrich, Germany) and 25 µg/ml kanamycin (Sigma-Aldrich, Germany) to a optical density (OD600) of 0.8. Recombinant protein expression was induced by adding 1 mM isopropyl β-D-thiogalactoside (IPTG; Roth, Germany) for 4 hours. *E. coli* cells were harvested by centrifugation at 3340 g for 10 min at 4°C. Bacterial pellets were then lysed in PBS (pH 7.4) containing 0.25 mg/ml lysozyme (Roth, Germany), 25 U/ml benzonase nuclease (Novagen, Germany), 10 mM imidazol (Roth, Germany), 1 mM PMSF (Sigma, USA) and 2 µM β-mercaptoethanol (Sigma, USA). Subsequently, bacterial lysates were sonicated 6 times (Bandelin Sonorex, Germany) on ice for 10 seconds each with >10 seconds rest and stored at −20°C. The rKLO8 was expressed as 6× His-tagged His-rKLO8 fusion protein and was recovered in the soluble fraction of the bacterial lysate by SDS-PAGE. Purification was carried out using nickel nitrilotriacetic (Ni-NTA) columns (Qiagen GmbH, Germany). The supernatant was loaded into a Ni-NTA column, which was pre-equilibrated with PBS, pH 7.4, containing 10 mM imidazole, 1 mM PMSF and 2 µM β-mercaptoethanol. The recombinant protein was eluted with the same buffer containing 400 mM imidazole. Salts and imidazole were removed by dialysis in PBS buffer. Protein concentration was determined using the Bradford assay compared to bovine serum albumin (BSA) as standard. Protein aliquots were kept frozen at −80°C.

### Bioinformatics analyses

The deduced amino acid sequence of the plasmid insert, determined with the ExPASy Proteomics Server of the Swiss Institute of Bioinformatics (http://web.expasy.org/translate/), was compared with published sequences obtained from the National Centre for Biotechnology Information (http://www.ncbi.nlm.nih.gov/). Immunodominant repeats of KLO8 (294 AA), K39 (252 AA) and KE16 (155 AA) were aligned using the ClustalW2-Multiple Sequence Alignment program (http://www.ebi.ac.uk/Tools/msa/clustalw2/). Homology search was performed with BLASTP 2.2.1 in 25.07.2012. Two different clones were analysed and found to contain the same insert (883 bp). Tandem Repeats Finder (http://tandem.bu.edu/trf/trf.html) [32] was used to locate and display tandem repeats in DNA sequences.

### SDS-polyacrylamide gel electrophoresis (PAGE) and Western blot analysis

The recombinant protein rKLO8 was loaded on a 12% SDS-PAGE under denaturing conditions [33] using fractions of bacterial cell lysates or the purified protein and stained with Coomassie Brilliant Blue G250 (Merck KGaA, Germany). Proteins were transferred to a nitrocellulose transfer membrane (Whatman GmbH, Germany) using the Bio-Rad Semi-dry Trans-Blot at 200 mA for 1 hr. The membrane was blocked with 5% BSA (w/v) in 100 mM NaCl, 0.05% Tween 20 (v/v) and 10 mM Tris-HCl, pH 7.4 (blocking buffer) and subsequently incubated for 18 hrs at 4°C with sera from patients – or healthy controls, diluted 1∶1000 in blocking buffer. After washing, blots were incubated for 1 hr at room temperature (R/T) with Peroxidase-conjugated Donkey Anti-Human IgG (H+L) (Jackson Immunoresearch Laboratories, USA) diluted 1∶10000. The protein bands were revealed with Maximum Sensitivity Substrate system (Thermo Scientific, USA).

### The recombinant protein rK39

The recombinant lipoprotein antigen rK39 of *L. infantum* (synonymous *L. chagasi*) was purchased from Rekom Biotech, S.L., Granada Spain. It contains repetitive immunodominant epitopes of kinesin-related protein. It was expressed as 6× His-tagged His-rK39 fusion protein at the C-terminus of the kinesin-related protein of *L. chagasi* with 100% identity with the accession number AAA29254.1. Upon receipt, the protein concentration was verified with the same method used to measure the recombinant protein rKLO8 (Bradford). Aliquots were kept at −80°C.

### ELISA

The optimal protein concentration and serum dilutions were determined using pooled sera from 10 VL patients from Sudan and 10 control sera from non-endemic areas in Sudan. To select conditions which best discriminate between positive and negative sera, different protein concentrations were titrated against serial dilutions of positive or negative sera. High protein-binding capacity polystyrene 96 ELISA plates (NUNC TM Serving Life Science, Denmark) were used. Protein concentrations of 5 ng/well to 50 ng/well were tested for coating ELISA plates overnight at 4°C in 0.1 M NaCO_3_ buffer, pH 9.6. Plates were washed with PBS containing 0.05% (v/v) Tween-20 and then blocked with 3% (w/v) BSA, in the same buffer, at R/T for 1–2 hours. After additional washes, 50 µl diluted positive or negative serum samples were added to each well, and plates were incubated at R/T for 45 minutes. After washing, 50 µl/well Peroxidase-conjugated AffiniPure Donkey Anti-Human IgG (H+L) (Jackson Immunoresearch Laboratories, USA), diluted 1∶10000, were added to each well and plates were incubated at R/T for further 1 hr. The reaction was visualised with hydrogen peroxide and tetramethylbenzidine (R&D Systems, USA). The reaction was stopped with 2N sulfuric acid after 10 minutes incubation in the dark. The optical density (OD) was measured at 450 nm using an ELISA microreader (FLUOstar Omega, BMG LABTECH). Each sample was tested in duplicates and the mean was taken. Samples reaching invalid or inconsistent results were repeated. As control, the pooled positive and negative sera were included in each plate, when testing individual sera.

### rK39 strip test

Individual IT LEISH dipstick kits using the recombinant K39 antigen [10] for detection of human visceral leishmaniasis antibodies were purchased from Bio-Rad, France. The test was performed and interpreted as recommended by the manufacturer. Sera were considered positive when a dark purple control band appeared. Samples with invalid results were repeated.

### Direct agglutination test, DAT

The DAT (ITMA-DAT/VL) kits (Lot 11D1B1) were purchased from the Institute of Tropical Medicine, Antwerp-Belgium (ITMA). The antigen is a freeze-dried suspension of trypsin-treated, fixed and stained promastigotes of *L. donovani* strain 1-S [34], [35]. The test was performed in 96 V-shape microplates (Greiner Bio One, Germany) according to the manufacturer's instructions. Besides internal controls, positive and negative pooled sera were included in each plate and results were read after overnight incubation at R/T. Samples with titres of 1∶≥3200 serum dilutions were considered positive, whereas samples with titres of 1∶800 and 1∶1600 serum dilutions were considered as borderline and were repeated.

### Statistical analysis

Data were analyzed using the GraphPad Prism software (GraphPad Prism Inc., San Diego, Ca). Significance of antibody responses was assessed using *Student t* test or one way *ANOVA* test. Differences of *p* values <0.05 were considered significant. Cut off values for each recombinant protein were defined as mean absorbance values of 30 sera of healthy controls from Sudan plus 3 standard deviations (SD). Sensitivity, specificity, positive predictive value (PPV) and negative predictive value (NPV) were calculated to assess usefulness of the diagnostic assays at 95% confidence intervals [36].

### Accession numbers

Nucleotide and amino acid sequences of *L. donovani* KLO8 have been deposited in GenBank under the accession numbers KC788285 and AGL98402, respectively. Accession numbers of other *Leishmania* kinesins were L07879.1, L07879.1, AAA29254.1, AAT40474.1, ABI14928.1, ADR74368.1 and AY615886.1.

## Results

### Molecular characterization of *KLO8*

**Figure 1:**
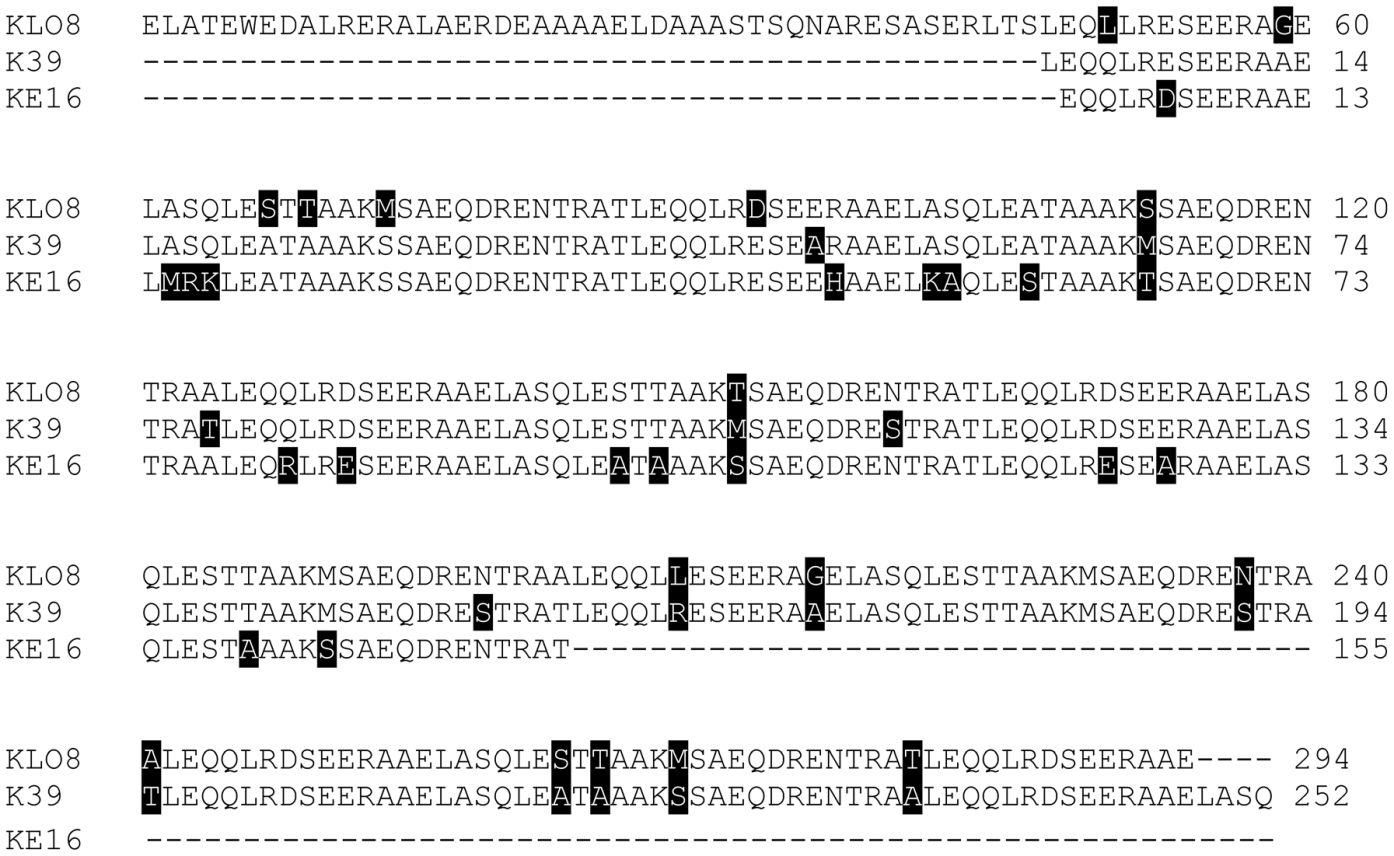
Protein sequence alignment. Immunodominant repeats of KLO8 (294 AA), K39 (252 AA) and KE16 (155 AA) were aligned using the ClustalW2-Multiple Sequence Alignment program. Different residues were highlighted in black and identical were left unmarked. Dashed lines indicate gaps.

A plasmid encoding a partial gene of the immunodominant kinesin protein *L. donovani* (*KLO8*) was constructed and subsequently subcloned to generate the expression vector pQE41/KLO8. Sequence analysis of the cloned fragment revealed a partial open reading frame (lacking the ATG) of a 883-basepair product. Tandem repeat analysis identified one repeat of 117 bp with 6.3 copies encoding 39 AAs. The DNA construct was further confirmed by restriction analysis using *BamH* I and *Sal* I resulting in an approximately 883-bp product. *KLO8* encodes a protein of 294 amino acids with a predicted molecular mass of 32.4 kDa and an isoelectric point (IP) of 4.39. Homology search in the protein database showed that KLO8 contains putative conserved domains of high similarity with kinesin proteins of *Leishmania*. KLO8 exhibited 93% and 88% amino acid identities with the kinesin proteins K39 of *L. infantum (synonym. L. chagasi)* strain BA-2 from Brazil (GenBank: AAA29254.1) and KE16 of *L. donovani* strain KE16 from India (GenBank: AAT40474.1), respectively. Of interest, KLO8 exhibited 97% identity with the kinesin protein Ldk39 of *L. donovani* 1S-CL2D from Sudan (GenBank: ABI14928.1), which however was never processed for development of a diagnostic test. Moreover, BLAST analysis showed 79% identity with the K28 fusion protein (GenBank: ADR74368.1), a synthetic protein construct derived from *L. donovani* [37]. The 756 bp immunodominant repeats of K39 (GenBank L07879.1) contains 6.4 copies of 117 bp encoding 252 AA. In contrast, KE16 (GenBank AY615886.1) showed only 4 copies of the 117 bp repeat encoding 155 AA. To identify differences in the immunodominant epitopes in the three *Leishmania* antigens, KLO8 was aligned with the AA repeats of K39 and KE16 using ClustalW2-Multiple Sequence Alignment (http://www.ebi.ac.uk/Tools/msa/clustalw2/. As shown in Fig. 1, immunodominant epitopes in the 3 antigens display presence of non-conservative amino acids. Differences in AA composition were highlighted in black and identical regions were left unmarked. These results confirm the variability in immunodominant repeats of *Leishmania* kinesin-related proteins.

### Expression, purification and immune recognition of the rKLO8 by VL patients' sera

**Figure 2:**
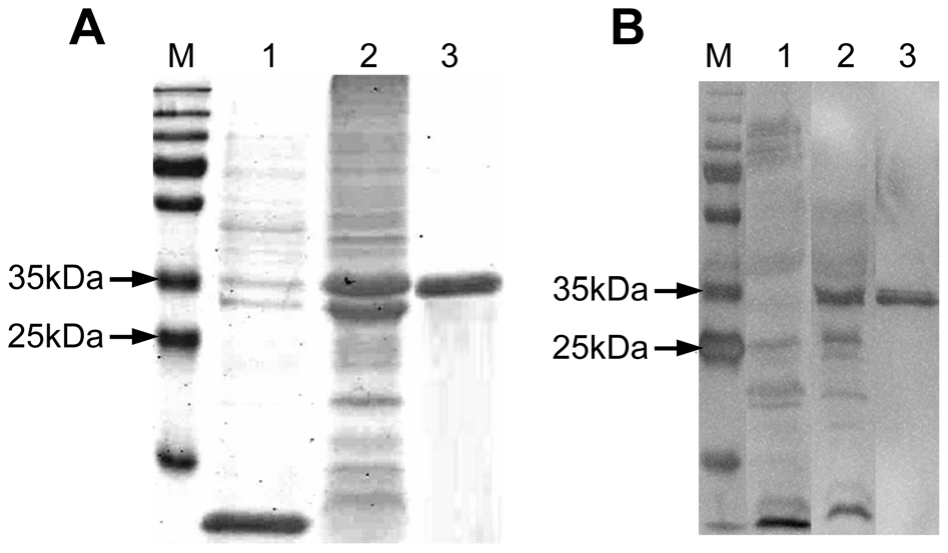
Expression and purification of the recombinant protein rKLO8. The *KLO8* gene was PCR amplified and cloned into the prokaryotic expression vector pQE41, expressed as 6× His-tagged fusion protein in *M15 E. coli* and purified on a Ni-NTA column. (A) Protein expression was checked on a 12% acrylamide gel stained with Comassie blue; lane 1 and 2, bacterial lysates from un-induced or 1 mM IPTG-induced cultures, respectively; lane 3, purified rKLO8 protein; M, Protein ladder. (B) Reactivity of the recombinant protein was confirmed in WB analysis using 10 pooled VL sera or 10 pooled healthy control sera from Sudan, diluted 1∶1000; lanes 1 and 2, lysates from IPTG induced cultures blotted with negative or positive sera, respectively; lane 3, purified rKLO8 blotted with positive sera; M, Protein ladder.

The KLO8 was expressed as His-tagged recombinant protein in *M15 E. coli* and expression was confirmed by SDS-PAGE. As shown in Fig. 2A (lane 2 & 3), the apparent molecular weight of the His tagged fusion protein was 35 kDa. The reactivity of purified recombinant protein rKLO8 was assessed in Western blot analysis using pooled sera from 10 VL patients or 10 healthy controls. As shown in Fig. 2B, the positive sera recognized the recombinant protein (lane 2 & 3), while the negative sera did not (lane 1). These results demonstrate that the recombinant protein rKLO8 is suitable for specific detection of *Leishmania* antibodies.

### rKLO8 ELISA

**Figure 3:**
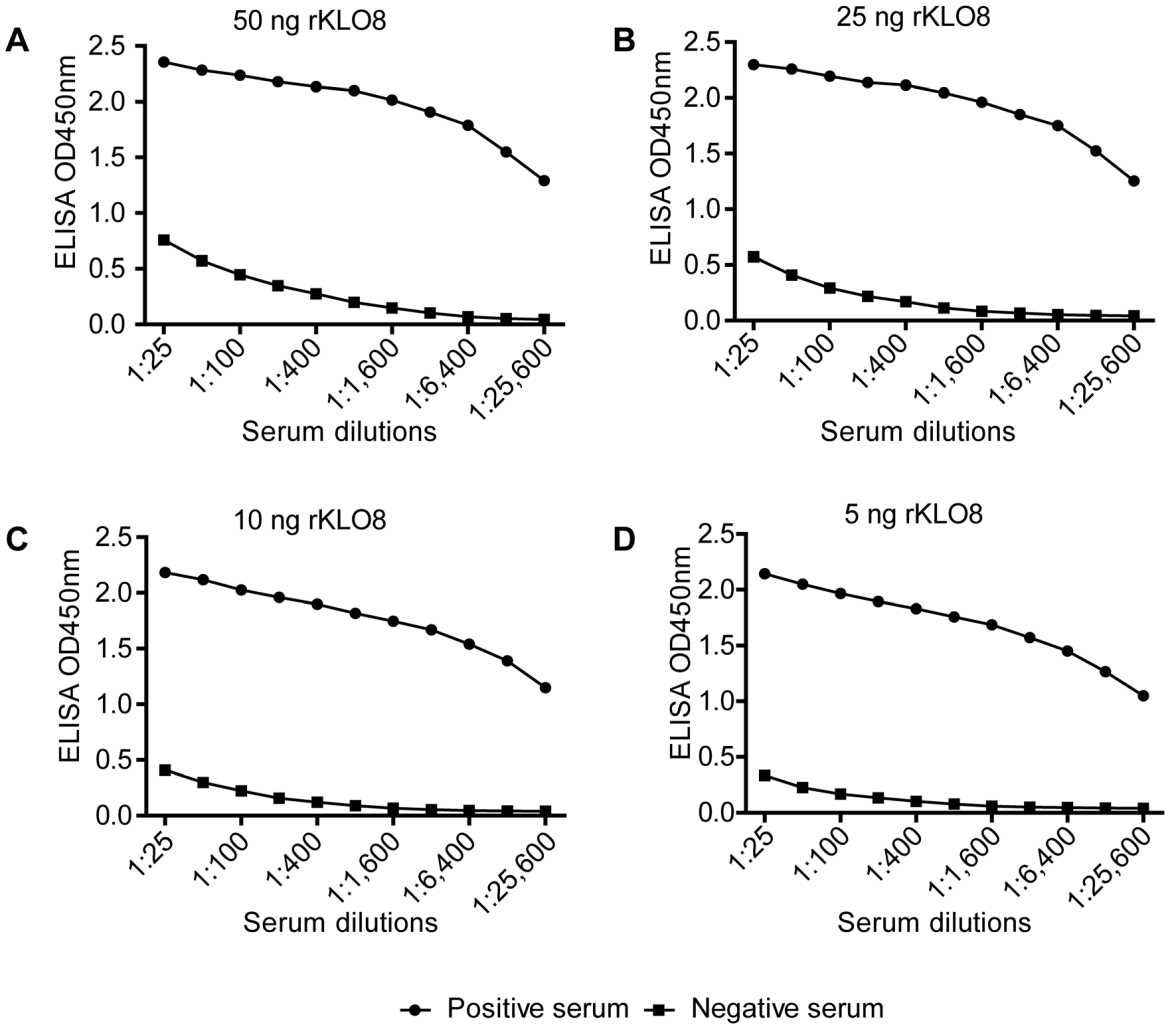
Establishment of an indirect IgG ELISA for specific detection of VL. For selection of the optimal ELISA conditions, 10 pooled VL sera or 10 pooled healthy control sera were titrated at serial twofold dilutions (1∶25–1∶25600) against different concentrations of the recombinant protein rKLO8. (A) 50 ng/100 µl, (B) 25 ng/100 µl, (C) 10 ng/100 µl, (D) 5 ng/100 µl. Sera were tested in duplicates and means were taken.

We next established an indirect IgG ELISA system using the recombinant protein rKLO8 for detection of *Leishmania*-specific antibodies in patients' sera. As shown in Fig. 3, all tested protein concentrations (50-5 ng/well) were recognized by pooled VL sera and did not cross-react with pooled sera from the healthy individuals. ODs of positive sera were at least 4 fold higher compared to negative sera, however this ratio changed to much higher values with more diluted sera. Coating ELISA plates with a concentration of 5 ng rKLO8 protein was sufficient for positive detection of sera from VL patients diluted up to 1∶25600. As a result of these titrations, a protein concentration of 5 ng/well and serum dilutions of 1∶800 were selected as standard conditions in subsequent experiments. In some experiments, VL sera with negative results at 1∶800, were re-tested at a serum dilution of 1∶100.

### Testing rKLO8 and rK39 for detection of IgG antibodies in VL patients from Sudan

**Figure 4:**
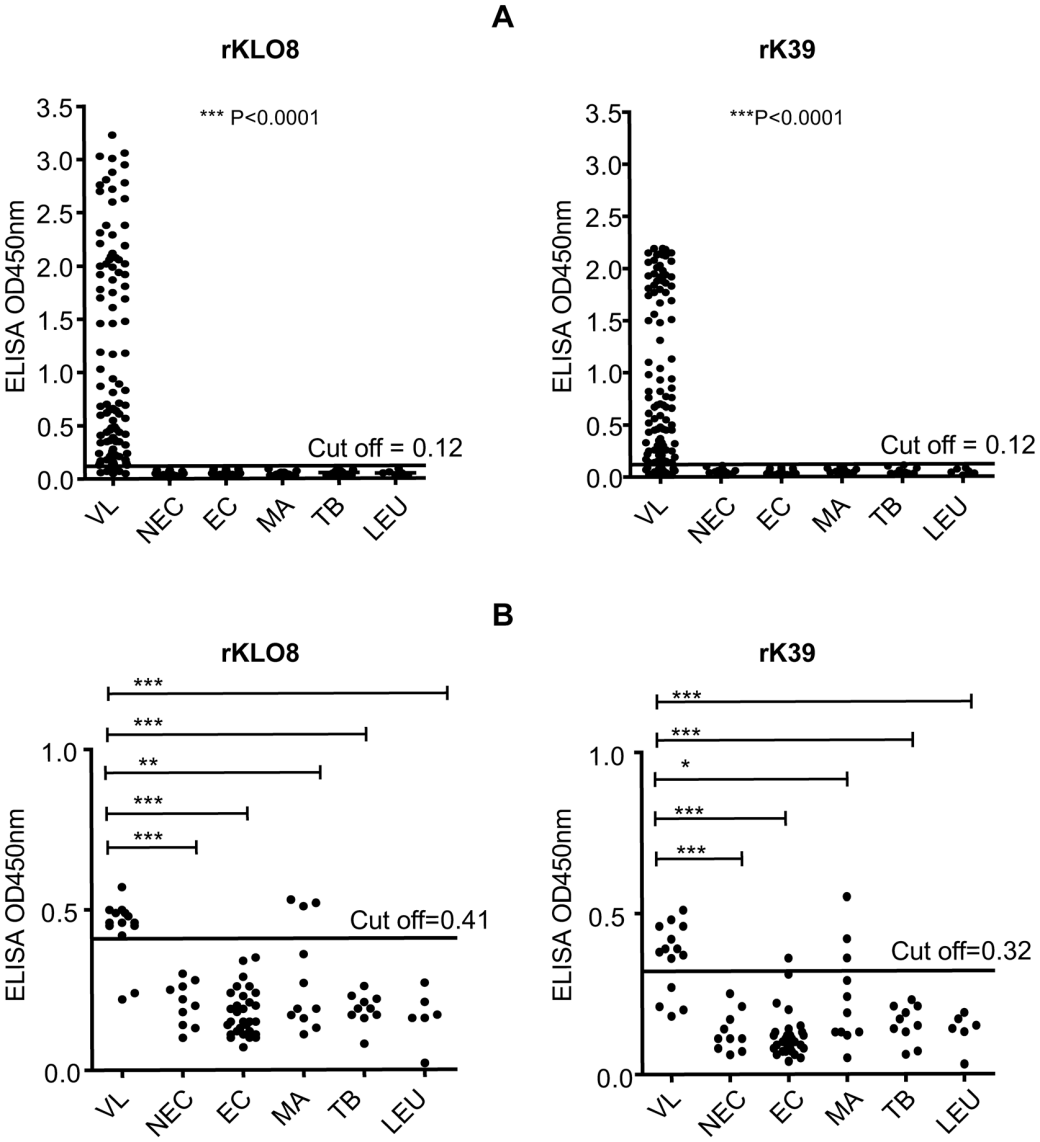
Comparative reactivity of *Leishmania* antibodies with rKLO8 and rK39. The rKLO8 or rK39 proteins were used and compared in ELISA using protein concentrations of 5 ng/100 µl in 0.1M sodium carbonate. A panel of sera from VL patients and controls were tested. Visceral leishmaniasis (VL; n = 106), non-VL controls (n = 77) including non-endemic healthy controls (NEC; n = 20), endemic healthy controls (EC; n = 30), malaria (MA; n = 11), tuberculosis (TB; n = 10), or leukaemia (LEU; n = 6). (A) Sera were tested at dilutions of 1∶800 and a cut off value (0.12) was established as means+3 SD of the OD measured for 30 healthy controls from Sudan. (B) VL sera (n = 14) with negative results at 1∶800 were re-tested at a serum dilution of 1∶100 and compared with the controls described in A. Cut off values were recalculated using 20 non-endemic healthy sera and found to be 0.41 and 0.32 for rKLO8 and rK39, respectively. Statistical Analysis was performed by *one way ANOVA* nonparametric test.

Reactivity of the two recombinant proteins rKLO8 or rK39 was evaluated in ELISA using individual human VL (n = 106) and control (n = 77) sera from Sudan. The recombinant protein rK39, obtained from Rekom Biotech, was expressed as 6× His-tagged fusion protein in *E. coli*. To ensure similar conditions, rKLO8 was also expressed as 6× His-tagged protein in *E. coli*. Sera of patients were diluted at 1∶800 and tested on a protein concentration of 5 ng/well (Fig. 4A). Quantitative analysis of antibodies in VL sera to both recombinant proteins demonstrated significantly higher antibody levels than those of control subjects (P<0.0001) although absorbance values among the patients' sera varied depending on the recombinant proteins. In general, sera tested on rKLO8 yielded higher OD values than on rK39 with a mean value of 1.12±0.97 for rKLO8 and 0.93±0.77 for rK39. In addition, sensitivity of rKLO8 was also increased with 92.5% (98/106) for rKLO8 versus 86.8% (92/106) for rK39. Notably, none of the healthy or diseased controls (n = 77) showed cross-reaction with either of the recombinant proteins (Fig. 4A). VL sera that were negative on rK39 or rKLO8 (n = 14) were then compared to control sera (n = 77) at 1∶100 serum dilution. As shown in Fig. 4B, re-testing on rKLO8 yielded increased positive detection of VL patients (12/14) as compared to rK39 (10/14) at cut-off values of 0.41 and 0.32 for rKLO8 and rK39, respectively. In addition, control sera tested on rKLO8 revealed less cross-reactivity as compared to rK39. Both proteins showed cross-reactivity with 3 sera from malaria patients and in addition rK39 showed false positivity of one healthy endemic individual.

### Diagnostic efficiency of rKLO8- and rK39 ELISA in comparison to two commercial kits

**Figure 5:**
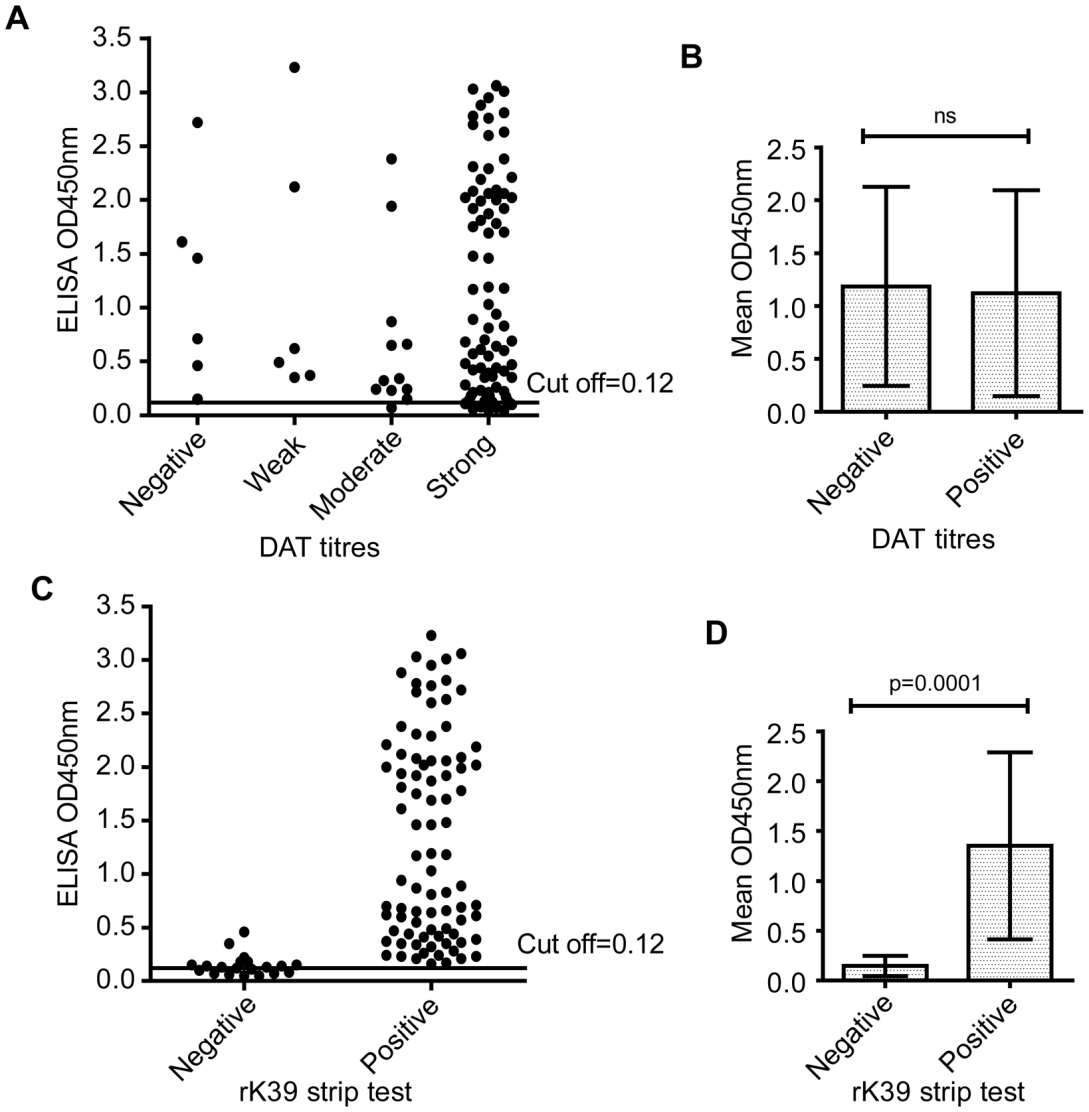
Comparison of reactivity for *Leishmania* antibody detection by rKLO8 ELISA, DAT and rK39 strip test. ODs for 106 VL sera diluted 1∶800 were measured in the rKLO8 ELISA and compared with DAT antibody titres or strip test results. (A) Sera were divided into 4 groups based on DAT titres; negative, 1∶±1600; weak, 1∶3200–1∶6400; moderate, 1∶12800–1∶25600; strong, 1∶≥51200. (B) Mean ODs for VL sera with negative or positive DAT titres were compared. Results are expressed as mean ± SD. *P value* (*Mann-Whitney U-test*). (C) OD values for VL sera with negative or positive strip test results were compared. (D) Mean ELISA OD values for VL sera with positive or negative strip test results. Dots represent values for individual sera and horizontal lines represent cut-off values.

Using the same panel of VL and control sera, the results obtained from the rKLO8 and rK39 ELISA were next compared with two commercial diagnostic kits, the rK39 strip test (Bio-Rad) and a freeze-dried version of DAT (ITMA-DAT/VL). As shown in Table 1, the overall sensitivities of rKLO8 (98.1%) and rK39 (96.2%), measured by ELISA, were higher than those of the rK39 strip test (81.1%) and DAT (94.3%). With respect to specificity, the rKLO8- and rK39 ELISA showed equally high performance (96.1% and 94.8%, respectively) but was slightly lower than DAT (100%) and rK39 strip test (98.7%). Accordingly, the PPVs and NPVs were 97.2% and 97.4% for the rKLO8 ELISA, 96.2% and 94.8% for the rK39 ELISA, 98.9% and 79.2.9% for the rK39 strip test and 100% and 92.8% for the DAT, respectively. Interestingly, results of the four tests showed some discrepancies. Although tested positive in the rKLO8 ELISA, 6 (5.7%) sera of the confirmed VL patients were negative (1∶<1600) in DAT (Fig. 5A, Table 1). In addition, sera of 6 patients had weak DAT titres (1∶3200–1∶6400) (Fig. 5A). On the other hand, while being positive in DAT, 4 (3.8%) or 2 (1.9%) sera of VL patients were not detected by rK39 or rKLO8, respectively (Table 1). Those 4 cases were also negative in the strip test. However, VL sera with positive or negative DAT results reacted similarly with rKLO8 (Fig. 5B), suggesting that rKLO8 or DAT monitor different immune reactivities. Thus, a combination of both rKLO8 ELISA and DAT provides 100% sensitivity for detection of VL. In addition, the rKLO8 ELISA detected all VL sera that were positive in the rK39 strip test (Fig. 5C), but sera negative in the strip test displayed still low antibody reactivity when tested with rKLO8 (p<0.0001) (Fig. 5D).

**Table 1:** Diagnostic performance of rKLO8 – and rK39 ELISA, rK39 strip test (ST) and DAT for visceral leishmaniasis in Sudan.

Serological test	TP	FN	TN	FP	Sensitivity (n = 106)	Specificity (n = 77)	PPV	NPV
					Estimated values at 95% CI			
rKLO8 ELISA	104	2	74	3	98.1%	96.1%	97.2%	97.4%
rK39 ELISA	102	4	73	4	96.2%	94.8%	97.2%	94.8%
rK39 ST (Bio Rad)	86	20	76	1	81.1%	98.7%	98.9%	79.2%
DAT (ITMA)	100	6	77	0	94.3%	100%	100%	92.8%

*Abbreviations*: TP, true positive; FN, false negative; TN, true negative; FP, false positive; PPV, positive predictive value; NPV, negative predictive value; CI, confidence interval; DAT, direct agglutination test; ITMA, Institute of Tropical Medicine Antwerp.

ELISA values were calculated combining results obtained at serum dilutions of 1∶800 and 1∶100. Detection of *Leishmania* in lymph node smears was used as reference. Specificity was calculated using 77 confirmed negative sera, including healthy controls, malaria -, TB-, and leukemia patients.

## Discussion

Despite the availability of several recombinant proteins for serodiagnosis of VL, commercially available rapid tests are still based mainly on the rK39. While these tests are quite effective in diagnosing VL in Brazil and Indian subcontinent, their use in East-Africa is not satisfactory. Despite the development of freeze dried DAT test based on Sudanese *L. donovani*, there is little interest in developing new rapid tests for VL in East Africa [38]. Improving VL diagnosis in these countries requires identification and testing of new antigens from autochthonous strains of *Leishmania*. Here, we aimed to clone, express and test a new recombinant protein of Sudanese *L. donovani* termed rKLO8 that shows homology with kinesin proteins of *Leishmania*. As expected, rKLO8 shows high sequence identity with the LdK39 protein of *L. donovani* 1S, a strain from Sudan which however has never been further used for a diagnostic procedure. Sequence analysis of KLO8 confirmed that the AA compositions of the immunodominant kinesin proteins of *Leishmania* show variability even among strains from the same region [39], [21]. The heterogeneity of kinesin immunodominant epitopes may explain why the use of rK39 and rKE16 is not sufficient to provide reliable diagnosis in the different endemic regions. Recently, the genetic diversity in the immunodominant kinesin repeats in strains of *L. donovani* and *L. infantum* has been documented [40].

Diagnostic methods with improved sensitivity for VL in Sudan are needed to replace low sensitive tests based on the kinesin of *L. chagasi* (rK39). Thus, a new rapid test based on the recombinant K28 protein has been developed and first data show promising results concerning serodiagnosis of VL patients in Sudan [37]. Our results with rKLO8 show also increased reactivity with patients sera as compared to rK39 ELISA. In addition, we (unpublished data) and others [37] have shown that some VL patients from Sudan have decreased immune responses to rK39, which explains the low sensitivity of rK39-based diagnostic tests in this region. This is in accordance with our finding that VL sera with negative rK39 strip test results show low but significant immune responses to rKLO8 (Fig. 5C and 5D). Thus, the increased reactivity of rKLO8 may provide enhanced detection of VL sera with low antibody titres. Here, we also show that the rKLO8 ELISA is more sensitive than the DAT (94.3%) and rK39 strip test (81.1%) confirming the low sensitivity of rK39 strip test in Sudan. Our data also show that difficulties of VL diagnosis in certain geographical areas might only be overcome, if the detection system is based on antigens derived from autochthonous parasites originating from the same endemic area [41], [42].

Antigenic variation due to parasite diversity has been proposed to be the cause for the low diagnostic sensitivity of VL diagnostics based on rK39 [20], [21]. In addition, the complexity and specificity of the humoral immune response against *Leishmania* parasites plays a crucial role in determining the outcome of serological assays. As a result, specific immune responses against *Leishmania* may be lost completely when tested against parasites isolated from different endemic area [43]. However, it cannot be generalized that antigens of endemic VL strains always result in improved diagnostic sensitivity, as sera of VL patients from Bangladesh reacted equally well with rK39 and rKRP42 derived from *L. donovani* from Bangladesh, despite marked heterogeneity between the two proteins [44].

An ideal diagnostic test should identify all positive sera without cross-reacting with negative sera. Our data show that none of the serological test used was able to detect all VL cases from Sudan. Only the combination of rKLO8 ELISA and DAT resulted in 100% diagnostic sensitivity. As antibodies of different specificities are detected, we recommend to combine rKLO8 ELISA and DAT to overcome the lower sensitivity in Sudan. Combination of different tests has also been suggested to overcome the problem of low sensitivity in East Africa [20], as detection of immune responses directed against different antigens is expected to improve sensitivity of an assay [43].

We have to be aware that recently infected persons have elevated IgM responses but not yet mounted an IgG response. Sera of such patients would give false negative results, if tested in an ELISA based on detection of IgG antibodies. This could explain the results of 2 patients that were tested negative in the rKLO8 ELISA and strip test despite strong positivity in DAT, which detects different antibody subclasses. More difficult to interpret are those 6 confirmed VL cases which were negative in DAT despite detectable antibody responses to rKLO8. Again, antibody specificities and parasite diversity could play a role. In addition, low antibody titers of these patients may result from coinfection with HIV, which complicate VL diagnosis by serological tests [45].

Unfortunately, malaria and other diseases are common in VL endemic region of Africa and Asia [46], [47]. Thus, a good test system needs robust discrimination between VL and potential co-infections. Indeed, malaria is known to be a major cause of cross reactivity to rK39 [48]. Cross reactivity to rK39 has also been reported with healthy sera of endemic and non-endemic controls from Sudan [37]. Our data indicate that a serum dilution of 1∶800 provides optimal specificity and sensitivity for rKLO8. Sera of malaria patients did not give a signal in the rKLO8 ELISA. This is in accordance with a previous study from Sudan, where 100% specificity of VL detection has been shown with sera tested at dilutions of 1∶1600 [30]. However in general, results with low antibody titres should be interpreted with caution. Notably, the DAT kit showed no cross reaction with any of the control sera tested and thus provides best specificity.

In conclusion, rKLO8 is a novel recombinant protein of *L. donovani* with increased reactivity to VL sera from Sudan. To finally evaluate its performance, rKLO8 has to be formulated as rapid test and assessed in a comparative field study.

## Supporting Information

Figure S1 Flow diagram of evaluating rKLO8 and rK39 in ELISA in comparison with two commercial tests of rK39 and DAT.
(TIF)

Checklist S1 STARD checklist for reporting of studies of diagnostic accuracy.
(DOC)
