# Supplementary figures and images for: rKLO8, a Novel Leishmania donovani – Derived Recombinant Immunodominant Protein for Sensitive Detection of Visceral Leishmaniasis in Sudan
Source: PLoS Negl Trop Dis. 2013 Jul 18;7(7):e2322. doi: 10.1371/journal.pntd.0002322 (PMC3715527; doi:10.1371/journal.pntd.0002322)

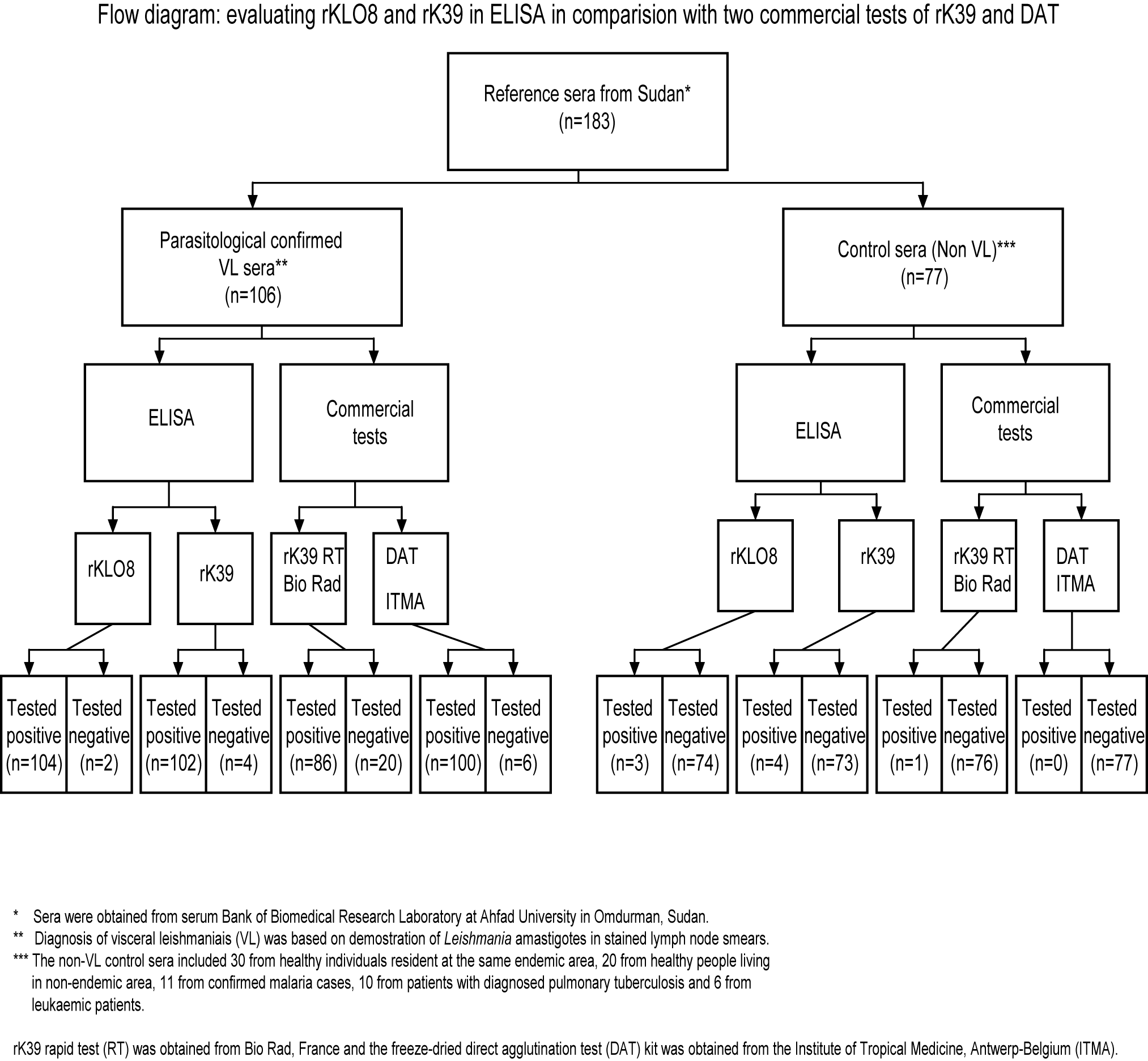

Supplement: Figure S1 — Flow diagram of evaluating rKLO8 and rK39 in ELISA in comparison with two commercial tests of rK39 and DAT. (TIF) [file pntd.0002322.s001.tif]
